# Myocardial Strain and Strain Rate in Kawasaki Disease: Range, Recovery, and Relationship to Systemic Inflammation/Coronary Artery Dilation

**DOI:** 10.4172/2155-9880.1000432

**Published:** 2016-04-21

**Authors:** Benjamin Frank, Jesse Davidson, Suhong Tong, Blake Martin, Heather Heizer, Marsha S Anderson, Mary P Glode, Samuel R Dominguez, Pei-Ni Jone

**Affiliations:** 1 Department of Pediatrics and Section of Cardiology, Children’s Hospital Colorado/University of Colorado, Denver, USA; 2 Department of Biostatistics, Children’s Hospital Colorado/University of Colorado, Denver, USA; 3 Department of Pediatrics and Section of Infectious Diseases, Children’s Hospital Colorado/University of Colorado, Denver, USA

**Keywords:** Mechanics, Echocardiography, Aneurysm, Contractility, Procalcitonin, Myocardial edema

## Abstract

**Background:**

Kawasaki Disease (KD), a systemic vasculitis of medium sized vessels, is the most common cause of acquired heart disease among children in the developed world. Some KD patients demonstrate echocardiographic evidence of depressed myocardial mechanics. However, the incidence, etiology, and reversibility of abnormal mechanics in KD patients remain undefined.

**Methods and results:**

We retrospectively studied 41 KD patients and measured myocardial strain and strain rate by velocity vector imaging from pre-treatment and convalescent echocardiograms. Pre-treatment procalcitonin, C-reactive protein (CRP), and coronary artery z-scores were obtained in all patients and compared between the groups with preserved versus depressed acute phase mechanics. The change in mechanics between the acute and convalescent phases was also assessed. Patients with initially low longitudinal strain improved by the convalescent period (mean difference - 4.0%; p<0.005) with the greatest improvement occurring in patients with the lowest initial strain (−7.3%; p<0.05). Patients with higher initial strain did not change significantly by the convalescent period. Patients with lower longitudinal and circumferential strain demonstrated higher median procalcitonin levels (1.2 vs. 0.3 ng/mL; p<0.05 and 1.8 vs. 0.4 ng/mL; p<0.05 respectively) and a trend towards higher CRP, but no difference in coronary artery z-scores. Strain rate was not associated with inflammatory markers or coronary artery z-scores.

**Conclusions:**

The range of strain found in our cohort was large. Improvement in mean strain was driven primarily by patients with lower initial strain. Lower strain was associated with increased markers of systemic inflammation, but not proximal coronary artery changes.

## Introduction

Kawasaki Disease (KD), a systemic vasculitis of medium sized vessels, is the most common cause of acquired heart disease among children. Recent data indicate an incidence rate of ~20 per 100,000 children under 5 years old in the United States [1]. Coronary artery dilation or aneurysms, the most clinically significant complications of KD, are seen in approximately 20% of untreated patients and 3–5% of treated patients [2].

Prior to the development of intravenous immune globulin (IVIG) for treatment of acute KD, myocardial dysfunction also played a prominent role in disease pathophysiology. Decreased function was thought to stem from inflammatory myocarditis. Imaging and biopsy studies of KD patients in the pre-IVIG era demonstrated evidence of myocardial inflammation and edema in 25–80% of cases, depending on the diagnostic modality and patient characteristics [3–6]. More recently, a post-mortem study of patients who died during the acute phase of KD showed myocardial interstitial inflammatory infiltrate in 100% of explant hearts, suggesting that inflammation could be associated with severe disease [7]. In the post-IVIG era, only a small percentage of KD patients develop overt signs of cardiogenic shock, [8] and the great majority of KD patients demonstrate normal conventional echocardiographic metrics of cardiac function, such as ejection fraction (EF) and fractional shortening (FS) [9]. Still, some subclinical dysfunction can be detected in IVIG-treated patients using research echocardiography methods such as rate-corrected velocity of circumferential fiber shortening [10,11].

Myocardial deformation analysis via echocardiography or cardiac magnetic resonance imaging Speckle-Tracking based measurement of strain and strain rate is a useful tool to evaluate left ventricular (LV) systolic function [12–17]. Strain is a dimensionless index of change in myocardial length during the cardiac cycle and is presented as a percentage. Strain rate is the rate of change of length, calculated as the time derivative of strain. This methodology has the potential to elucidate, in many disease processes, subtle changes in myocardial mechanics and function not demonstrated by conventional measures [12,17,18].

Three recent studies have demonstrated lower (less negative) mean global longitudinal strain in cohorts of acute phase KD patients relative to controls [9,13,19]. However, all three studies found a wide range of strain values in the KD cohort as well as significant overlap with the control group. While one study [19] demonstrated recovery of strain by the convalescent period, it is unclear if the differences in deformation and subsequent recovery in these studies were uniform across the cohorts or driven by a subgroup with initially depressed mechanics. Further exploration is required to better define the variation of myocardial deformation within KD cohorts and to assess if variation in myocardial deformation may help identify subgroups with different pathophysiology.

To begin to resolve these unanswered questions, we designed a retrospective study in a cohort of acute KD patients to evaluate the range of myocardial deformation and changes over time in KD patients with lower versus higher initial myocardial deformation. Specifically, we hypothesized that acute KD patients would demonstrate a wide range of myocardial deformation and that patients with initially lower strain and strain rate would show increased (more negative) deformation by the convalescent phase identifying a subgroup with depressed initial deformation. On the other hand patients with initially higher deformation would demonstrate either no change or a decrease in deformation over the same time period. In addition, based on findings from prior studies [9,13,19] we hypothesized that patients with lower initial myocardial deformation would have elevated acute markers of systemic inflammation (C-reactive protein [CRP] and the more recently established procalcitonin) but no difference in proximal coronary artery dilation relative to those with higher initial deformation.

## Materials and Methods

### Subjects

We identified patients treated between December 2008 and January 2013 at Children’s Hospital Colorado for a diagnosis of acute KD. Patients were determined to have “complete” KD if they experienced five days of fever and a minimum of four out of five clinical criteria, as defined by Fimbres et al. [20]. The remainder of study patients (“incomplete KD”) included those with five days of fever and 2–3 clinical criteria plus either echocardiographic evidence of coronary artery disease or at least three supportive laboratory criteria. To meet inclusion criteria, patients were required to have a pre-treatment C-reactive protein (CRP), a pre-treatment stored blood sample available for procalcitonin (PCT) analysis, and a pre-treatment echocardiogram for mechanics analysis. Patients were excluded if missing any of the above, and specifically if the initial echocardiogram was done after IVIG treatment. Pediatric infectious disease specialists consulted on all patients during their hospitalization and confirmed the diagnosis of KD. Study data were collected and managed using REDCap electronic data capture tools hosted at University of Colorado. The Colorado Multiple Institution Review Board approved this study.

### Clinical data

Clinical data were collected for all patients including demographics, date of fever, clinical signs/symptoms, date and type of treatment, response to treatment, and need for intensive care admission. Laboratory data collected included CRP (peak and closest to treatment initiation, [mg/dL]), erythrocyte sedimentation rate (ESR, [mm/h]), white blood cell count, and liver function tests. In addition, PCT levels [ng/mL] were measured for all patients on stored, pre-treatment blood samples using a PCT sensitive Kryptor System (Brahms, Aktiengesllschaft, Annapolis, MD, USA).

### Echocardiographic analysis

Echocardiograms were obtained as part of routine clinical care. All echocardiograms were originally acquired using either a Phillips IE33 (Philips Ultrasound, Bothell, WA) or GE Vivid 7 (GE Medical Systems, Milwaukee, WI)) system and stored as Digital Imaging and Communications in Medicine (DICOM) images on AGFA Heartlab system (Mortsel, Belgium). For the purpose of this study, we analyzed acute phase echocardiograms recorded prior to intravenous immune globulin (IVIG) infusion and convalescent phase echocardiograms recorded during outpatient follow-up (~6 weeks after initial diagnosis). FS and EF were recorded for each patient from M-mode in the parasternal short axis view at the level of the papillary muscles, along with proximal right coronary artery (RCA), left main coronary artery (LMCA), and left anterior descending coronary artery (LAD) measurements and body surface area normalized Z-scores [21–23]. Left ventricular mass and mass index (indexed to body surface area, [g/m^2^]) were also assessed using standard M-mode echocardiography for use as an indirect measure of myocardial edema [24,25].

### Advanced myocardial mechanics analysis

**Figure 1:**
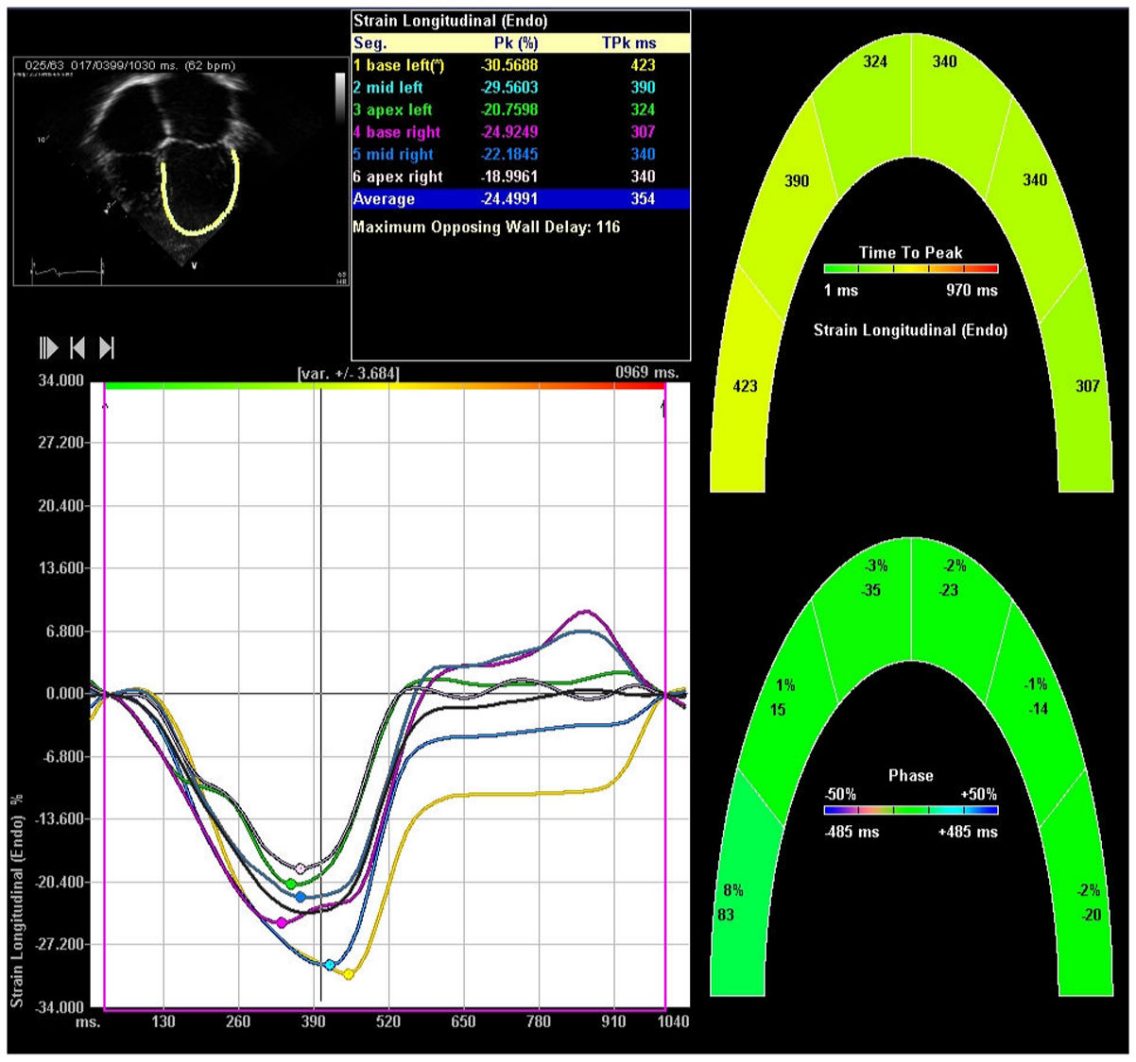
VVI longitudinal strain output.

Apical four-chamber and parasternal short axis images were utilized for analysis of speckle-tracking based myocardial mechanics (Figure 1). A single investigator (JD) clipped and extracted stored DICOM images for each patient and uploaded them into the Syngo velocity-vector imaging (VVI) software (Siemens, Germany). To prevent any potential bias, a separate investigator (BF) performed all speckle-tracking measurements. This investigator was blinded to details of the patients’ clinical course and all non-pertinent echocardiographic views, including those of the coronary arteries.

Endocardial tracking of the apical four-chamber view was employed to calculate systolic longitudinal strain and strain rate of the left ventricle. Endocardial and epicardial tracking of the parasternal short axis views at the level of the papillary muscle were utilized to calculate systolic circumferential and radial strain and strain rate. Reported strain and strain rate values represent the average of six tracked segments. Segmental data were not analyzed individually. A tracing was deemed acceptable when visual inspection and the VVI software indicated adequate tracking. If the VVI software was unable to track a single segment, that segment was excluded and the averaged value of the remaining five segments was taken. If more than a single segment was interpretable, data from that image were excluded from further analysis.

By convention, negative longitudinal and circumferential strain and strain rate values indicate shortening while positive values indicate lengthening, with more highly negative values representing better cardiac function. For ease of reporting we refer to more highly negative values as higher deformation and less negative values as lower deformation. Radial strain is reported in positive values, with higher radial strain equating to higher deformation. No consensus normal values exist for myocardial deformation in children. Therefore, in order to examine the subgroup with the lowest deformation, we divided the cohort into those with the highest 75% of values versus those with lowest 25% of values for each measurement (longitudinal/circumferential strain/strain rate). As an additional assessment, we also evaluated longitudinal strain against a best-available normal value from the cohort published by Marcus et al. [26]. For our predominant age group (1–9 years), this leads to a “normal” or “preserved” value for longitudinal strain of −18% to −24% [26]. Subjects with longitudinal strain lower than −18% were considered “depressed”.

### Statistical analysis

Continuous data were tabulated by mean and standard deviation or median and range as appropriate for the distribution of the data, while count and percent were used for categorical data. The distribution was assessed using the Shapiro-Wilk test with a p-value greater than 0.05 considered as normally distributed. Clinical characteristics and candidate predictors were compared between groups of dichotomized outcomes using a two-sample T-Test or Wilcoxon rank sum test. Changes in outcome between paired samples were compared by paired T-Test. Multiple logistic regression was employed to model the relationship between clinical outcomes and candidate predictors. Receiver Operating Characteristics (ROC) was performed to find the best cut-offs for PCT, CRP, LV Mass and coronary artery Z-scores. Spearman rank correlation test and scattered plot were used to demonstrate the correlations between longitudinal strain and circumferential strain. Kappa coefficients were calculated to assess the agreement between longitudinal and circumferential strain [27]. All the analyses and graphs were done with SAS V9.3 (SAS Institute Inc, Cary, NC).

## Results

### Patient demographics

Eighty-five patients with KD had the requisite pre-treatment CRP and stored blood for PCT analysis during the study time period. Of those, 41 patients also had a pre-treatment echo and were used in this analysis (Table 1). Mean age was 46.3 months, and ethnicity was representative of the population of the greater Denver area. There was no difference in age, gender, race, day of illness, or percent of cases with incomplete KD between the 41 included subjects and the 44 who were excluded based on the lack of a pre-treatment echo. A total of 81 echocardiograms (41 patients and two time points) were analyzed for circumferential, radial, and longitudinal strain. Four echocardiograms had one dimension of analysis excluded from final analysis because of unacceptable VVI tracings. No single echocardiogram had both the longitudinal and circumferential analysis excluded. Eight echocardiograms included in the final analysis had one segment of one dimension excluded due to poor tracking. The data from radial strain analysis was determined to be highly variable across all patients and no meaningful relationships were established with any other metric. EF was also measured for each patient. Forty patients demonstrated normal EFs (≥ 55%) compared to one patient with an abnormal EF (<55%). We found no significant correlation between longitudinal strain and EF (r=−0.14; p=0.39). No subject demonstrated regional wall motion abnormalities suggestive of acute myocardial infarction.

### Acute versus convalescent myocardial mechanics

**Figure 2:**
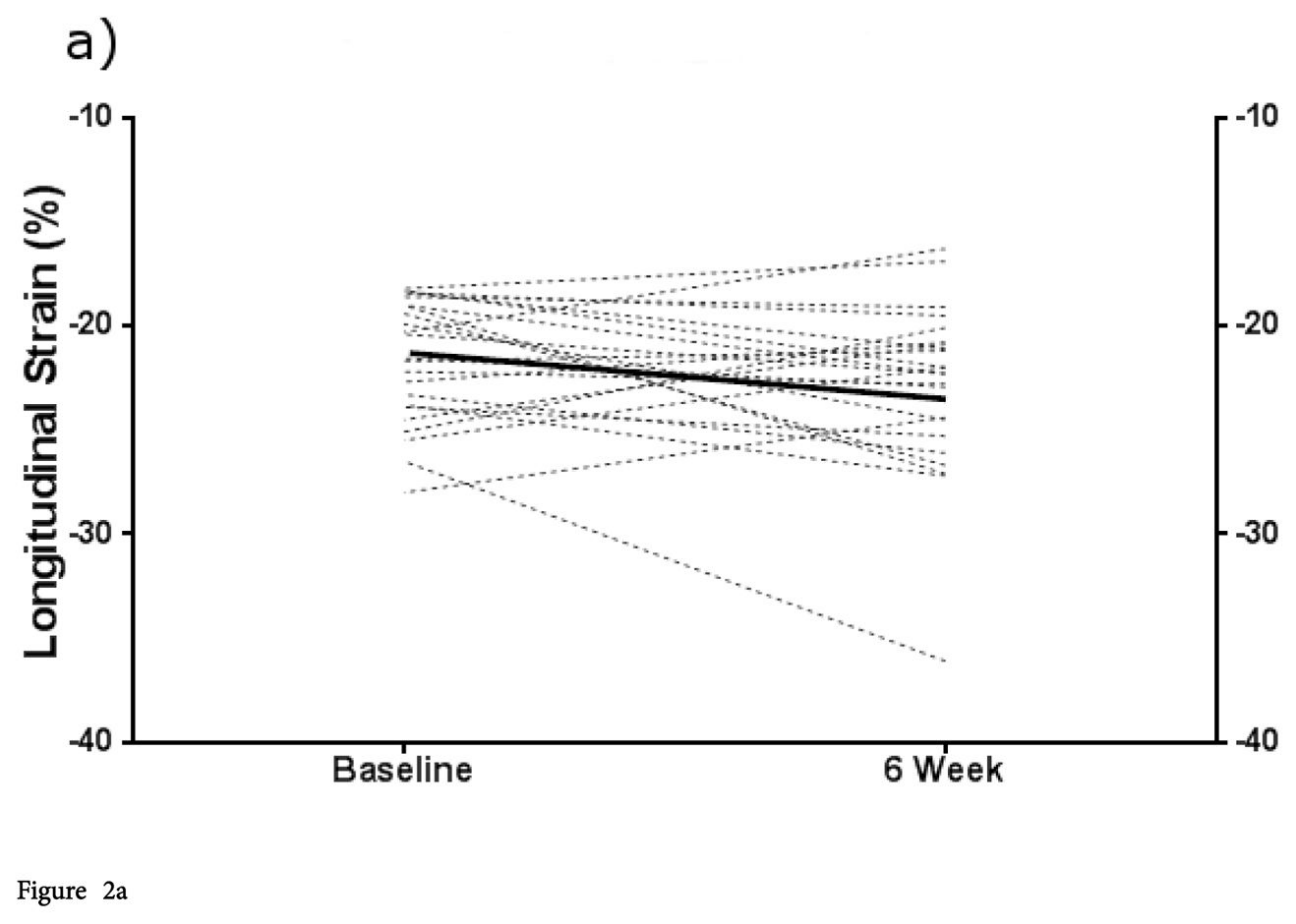

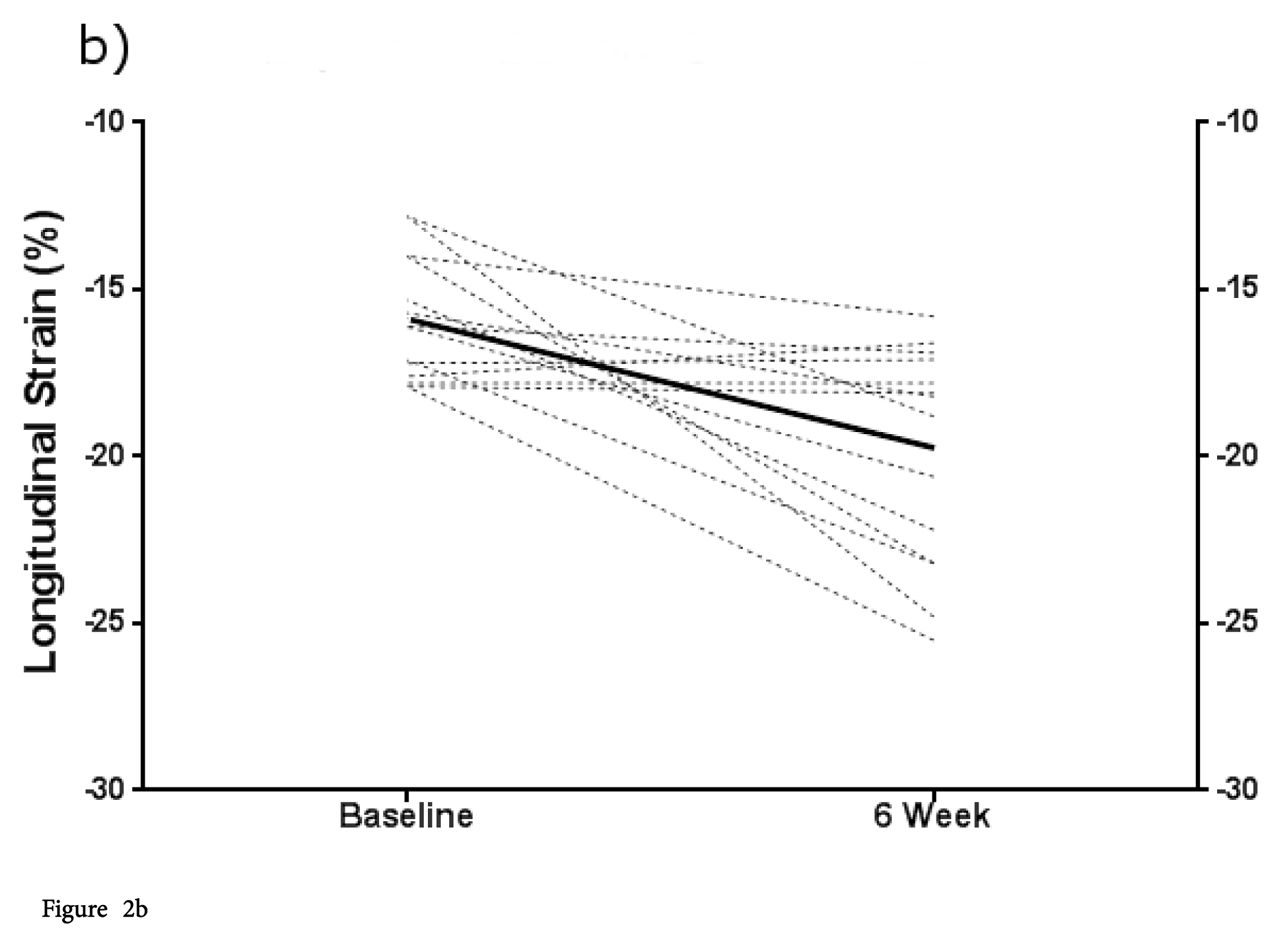

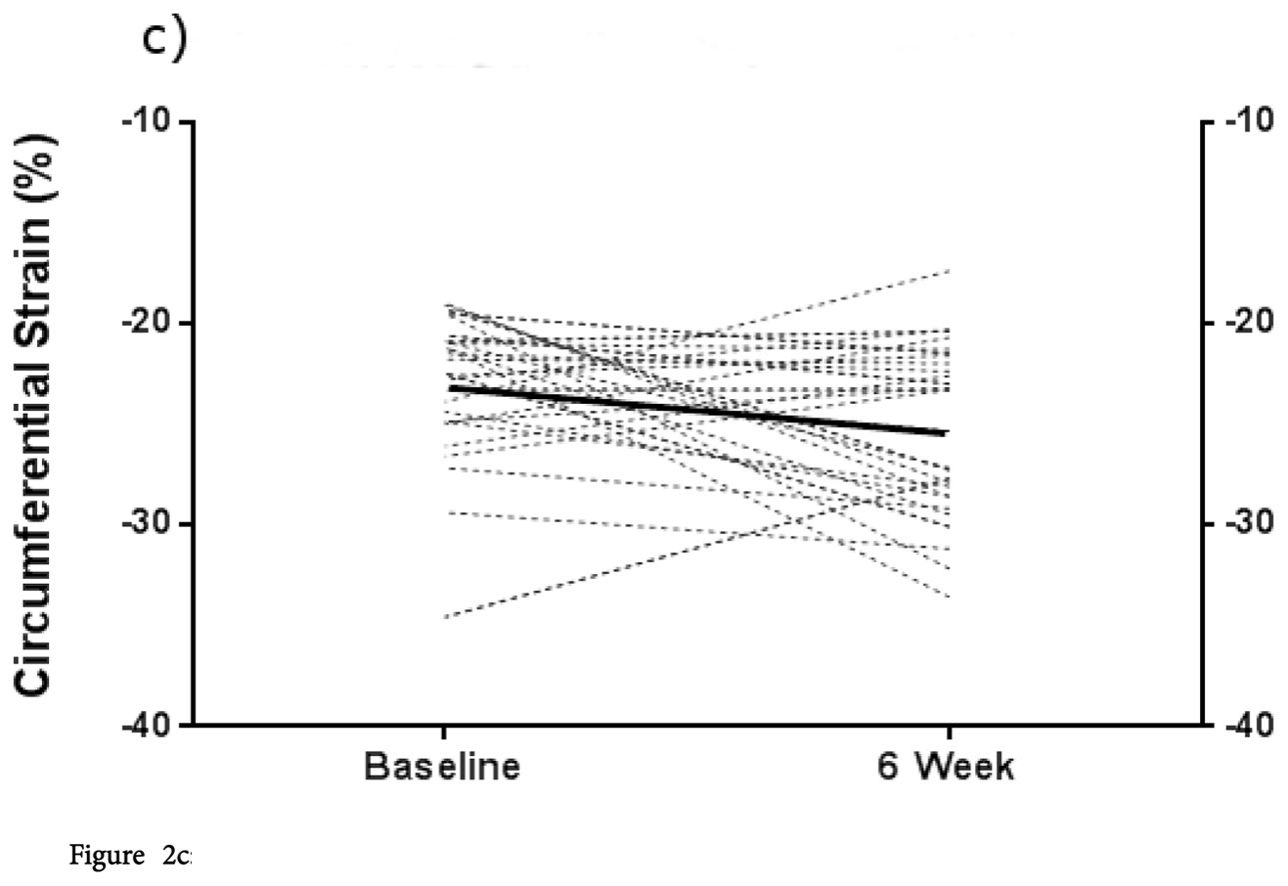

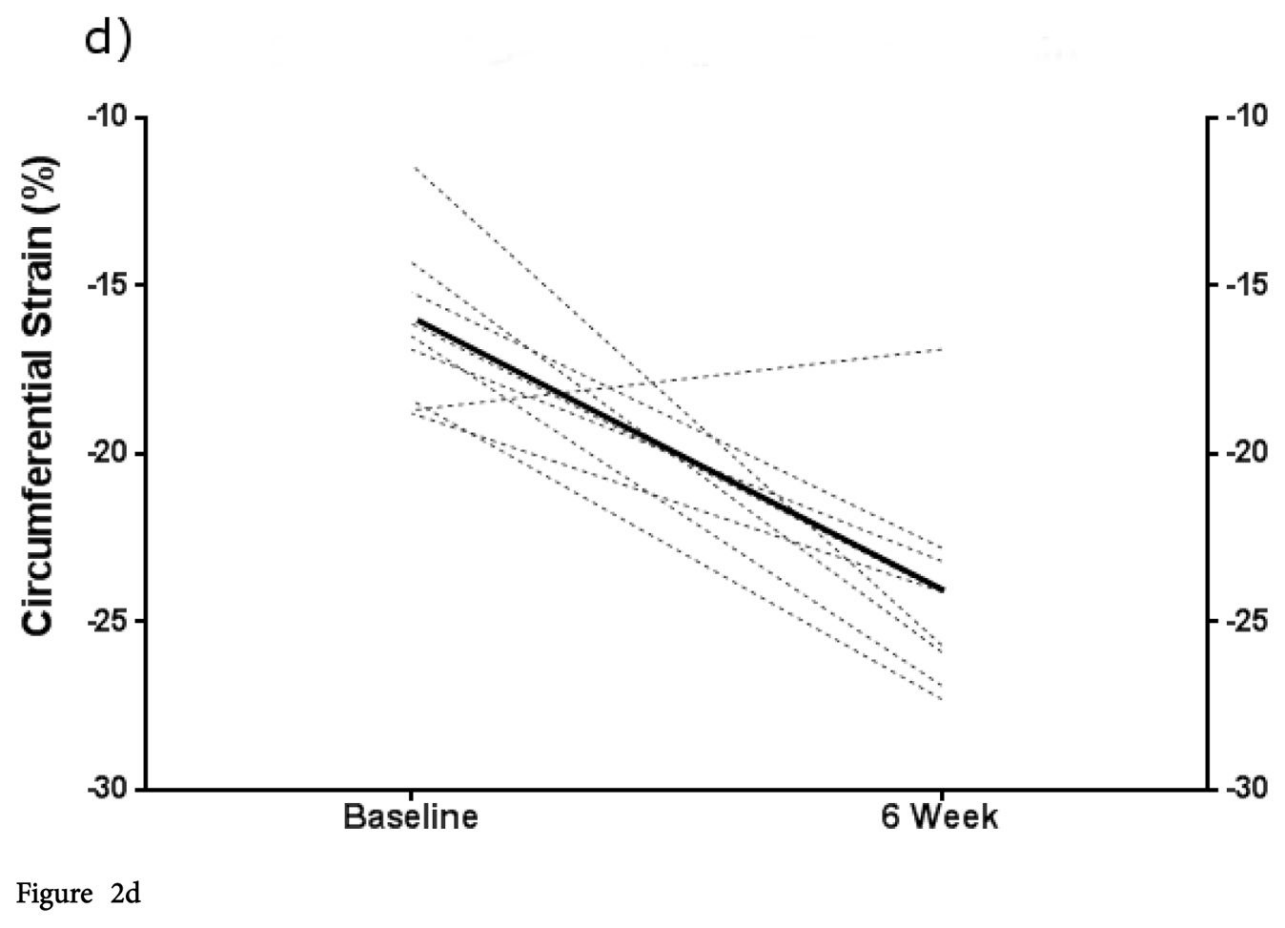
Figure 2a: Change in ε (longitudinal and circumferential) from acute to convalescent phase stratified by baseline longitudinal or circumferential ε. Baseline longitudinal ε ≤ −18 (normal or hyperdynamic). Figure 2b: Change in ε (longitudinal and circumferential) from acute to convalescent phase stratified by baseline longitudinal or circumferential ε. Baseline longitudinal ε≥18 (low). Figure 2c: Change in ε (longitudinal and circumferential) from acute to convalescent phase stratified by baseline longitudinal or circumferential ε. Baseline circumferential strain lower 25%. Figure 2d: Change in ε (longitudinal and circumferential) from acute to convalescent phase stratified by baseline longitudinal or circumferential ε. Baseline circumferential strain upper 75%. Bold lines represent mean values.

In our study population, mean longitudinal strain improved between the baseline and convalescent time points (mean difference −2.3%; p<0.005). This improvement in myocardial mechanics was not, however, equally distributed across all patients. Patients with depressed longitudinal strain at baseline showed statistically higher strain at convalescence than during the acute phase (−19.9% vs. −15.9% [mean difference −4%; p<0.005]), while those with preserved strain at baseline showed no significant change between these time points. Patients with the most severely depressed baseline strain (>−15%) demonstrated an even greater improvement (−13.4% to − 20.7% [mean difference −7.3%; p<0.05]). Changes for individual patients and mean differences are demonstrated in Figure 2. Comparison of baseline and convalescent circumferential strains followed a similar pattern. Patients with lower circumferential strain at baseline improved by 6 weeks; their mean convalescent value was not different from those patients with higher circumferential strain throughout.

### Myocardial mechanics and inflammation

Inflammatory biomarkers were compared in patients with lower strain were compared to the group with higher strain. The lower strain group showed higher median PCT compared to the higher strain group with borderline significance (1.3 ng/mL vs. 0.4 ng/mL; p=0.05). CRP also appeared higher in this group, but the difference did not reach statistical significance (11.0 mg/dL vs. 5.3 mg/dL; p=0.11). When assessed using best available normal longitudinal strain values (≤ −18% vs. >−18%) similar trends were seen with PCT being statistically significantly difference at this strain cut-off (Table 2). ESR and albumin were measured without clinically or statistically significant differences between the two groups. The two groups did not appear to differ in LAD, LMCA, or RCA Z-scores. No significant correlation between longitudinal strain and variations in EF was found. We also did not find a significant linear relationship between strain values and PCT or CRP. There was no difference in age between the patients with lower versus higher strain (median age 39.2 months versus 43 months; p=0.81), suggesting that this difference in strain was not likely to be due to age related differences in myocardial deformation.

To evaluate for a potential dose response relationship, we also assessed markers of systemic inflammation in groups with progressively more depressed strain (<−18% vs. −18 to −15% vs. >−15%) (Table 3). While the numbers in each subgroup precluded adequate statistical power, there is a similar trend towards increasing CRP and PCT with lower longitudinal strain.

Acute phase circumferential strain demonstrated a similar relationship to markers of systemic inflammation (Table 4). Patients with lower circumferential strain had significantly higher median PCTs than those with higher strain. Similarly, median CRP appeared higher in the patients with lower circumferential strain but this difference did not reach statistical significance. Lower circumferential strain was not associated with a difference in ESR, albumin, or LAD, LMCA, or RCA Z-scores.

Overall correlation between longitudinal and circumferential strain in individual patients was moderate (r=0.45; p<0.01). Although imperfectly correlated in magnitude, we found that patients with depressed mechanics measured in one dimension tended to have depressed mechanics in the other dimension (kappa=0.36), further suggesting a true decrease in overall function.

### Strain rate and inflammation

For longitudinal strain rate, a cut-off of −1.4%/s separated the upper 75% of patients from the lower 25%. No statistically significant findings were demonstrated when comparing longitudinal strain rate with PCT, CRP, or coronary Z-scores. Subjects were similarly grouped for analysis of circumferential strain rate (cut-off - 1.8%/s) and, again, no statistically significant associations were noted between circumferential strain rate and PCT, CRP, or coronary Z-scores.

### Predictors of myocardial mechanics

ROC curves were generated to model baseline PCT, CRP, LAD z-score, and cardiac mass index as predictors of longitudinal strain. While none were ideal predictors of which patients would have abnormal strain (areas under the curve ranged 0.62–0.72), PCT was the most accurate independent variable. A multivariate regression analysis was employed to further elucidate these relationships. No individual variable was an independent predictor of abnormal mechanics.

### Myocardial edema

LV mass index was measured for each patient in our study population as a possible indirect marker of myocardial edema. At baseline, patients with preserved and depressed longitudinal strain demonstrated average mass index values of 59.3 and 51.7 g/m^2^, respectively (p=0.11). Patients with elevated mass indices showed no differences in PCT (0.6 vs. 0.6, p=0.35), CRP (5.3 vs. 9.9, p=0.44), ESR (68.6 vs. 66.4, p=0.84), or coronary z-scores (0.0 vs. 0.1, p=0.86) compared with those patients with normal LV mass index. Similarly, patients with elevated mass index showed no difference in serum albumin measurements when compared with their normal mass index counterparts (3.4 vs. 3.55, p=0.74). At the convalescent time point, mass index values had decreased for all patients. This improvement was most significant for patients with the highest LV mass indices at baseline, such that the preserved and depressed longitudinal strain groups were statistically indistinguishable at 6 weeks (mean mass index 48.4 preserved vs. 47.5 depressed).

### Deformation in critically ill KD Patients

Four patients in our cohort were critically ill and admitted to the intensive care unit for hypotension and cardiovascular support. While the numbers of these patients in our cohort are too low to allow any meaningful statistical analysis we did collect qualitative information on their strain relative to inotropic and vasoactive support. Three patients demonstrated depressed strain even with substantial inotropic support (dopamine and/or epinephrine). The fourth initially displayed hyperdynamic strain and hypotension that was unresponsive to escalating dopamine therapy suggesting vasogenic rather than cardiogenic shock. This patient was subsequently transitioned from dopamine to norepinephrine with rapid resolution of shock.

## Discussion

Cardiac function in children with acute KD has been examined using a variety of measures [10,19,22,28–32]. Pre-IVIG era studies consistently demonstrated a high incidence of patients with abnormal left ventricular EF and FS [10,11,28,29]. In studies from the IVIG era, abnormal left ventricular EF and FS occur infrequently, suggesting that early diagnosis and treatment may reduce or eliminate significant myocardial dysfunction [10,11]. Efforts to find easy echocardiographic methods to evaluate for sub-clinical dysfunction in acute KD patients prior to IVIG treatment have met with mixed results [22,31].

Speckle-tracking based myocardial strain is capable of detecting early systolic dysfunction in multiple disease states, [12,20,33,34] but its role in KD is not well defined. Recently, three studies demonstrated depressed average myocardial strain in KD patient cohorts relative to controls [9,13,19]. However, the range and reversibility of these abnormalities remain poorly understood as are the associations with inflammation and proximal coronary artery changes. Our study is the first to compare KD patients with lower versus higher strain and strain rate in an effort to further characterize these subgroups of a KD cohort.

Our patients displayed a significant range of myocardial strain on the acute phase echo. Those patients with initially lower strain improved substantially by the convalescent echo, while those with higher strain showed much less change or even a lower strain value at the later time point. The results were similar when using either longitudinal or circumferential strain. These findings suggest that the lower average strain in KD cohorts seen on prior studies [9,13,18,19] is probably driven by a subgroup of the cohort with truly depressed initial strain. When comparing the longitudinal strain values to the best available normal cohort, [26] both hyperdynamic and depressed functions were observed within the cohort during the acute phase. Fifty-one percent of patients demonstrated normal strain by these criteria. A substantial portion of the patients demonstrated depressed strain (39%) using these best available normal values, while fewer patients had hyperdynamic function (10%). Interestingly, 3 of the 4 patients with initially hyperdynamic deformation by these parameters demonstrated a decrease or “normalization” in strain by convalescence. Conclusions regarding the incidence of abnormal circumferential strain in this population are difficult to make due to the lack of any normative data. However, the generally high agreement between depressed longitudinal and circumferential strain suggests that mechanics are globally depressed in this subset of patients.

In contrast to strain measurements, some differences exist in the literature regarding the effects of acute KD on myocardial strain rate. Yu et al. identified depressed strain but not strain rate in acute KD patients relative to controls, while McCandless et al. [9] found both strain and strain rate to be depressed in acute KD [9,13]. In our cohort, strain rate behaved differently than strain and was not found to be significantly associated with markers of inflammation. As strain rate is thought to possibly be a better predictor of inherent contractility than strain, [35–40] it is plausible that strain may be affected by interstitial inflammation/edema while strain rate may not be affected unless actual myocyte injury occurs. However, due to limited knowledge of normal strain rate values and difficulty obtaining accurate strain rate measurements on stored images, these results should be interpreted with caution.

The etiology of abnormal myocardial strain in acute KD remains incompletely defined. The results of our study provide some modest evidence in favor of an inflammatory etiology of depressed strain: we demonstrated a significant association between depressed strain and increased markers of systemic inflammation, this association was consistent across the longitudinal and circumferential dimensions, and we found a trend towards a dose-response relationship between strain and inflammation despite a small sample size. On the other hand, we did not identify a significant association between strain and proximal coronary artery dimensions. Our findings are consistent with a recent publication by Gaur, et al. [41] examining the differences in myocardial deformation among KD patients with and without echocardiographic markers of carditis (mitral regurgitation and pericardial effusion). The authors found a significantly lower strain in patients with mitral regurgitation and pericardial effusion, which improved by convalescence. Also similar to our study, Gaur, et al. found a modest correlation between inflammatory markers (CRP/ESR) and myocardial strain. Examining specific inflammatory markers in our study, PCT appeared to be marginally better at predicting altered strain than CRP although neither was sufficiently predictive to have clinical utility for this purpose.

Our findings are consistent with what is known about the biology of myocardial involvement in KD. In the pre-IVIG era, patients with abnormal EF and FS were thought to fall into two basic categories: those with abnormal wall motion due to large vessel occlusion/ischemia, and those with generalized hypokinesia presumed secondary to myocardial inflammation and edema [29]. In recent years, large vessel occlusion and myocardial infarction have become rare during the acute phase of KD due to heightened awareness of the disease and early IVIG treatment [35]. Severe global dysfunction, while uncommon, is still occasionally seen in acute KD and has been theorized to arise from several different mechanisms including interstitial edema (considered by some to be the characteristic myocardial lesion of KD), [3,7] inflammatory myocyte injury, [6,36] and microvascular inflammation/occlusion [6]. Biomarker studies in acute KD also show evidence of myocardial dysfunction including frequent mild to moderate elevations in NT-pro-BNP (a marker of ventricular volume or pressure overload) [36]. Interestingly, troponin I, a marker of myocyte injury, generally shows only minor elevations in acute KD [37] and is weakly correlated with markers of function (NT-pro-BNP), suggesting that direct myocardial injury is not the primary mechanism of cardiac dysfunction [36].

To further evaluate the potential association of dysfunction with myocardial edema, we examined the association between LV mass index and myocardial strain. Yu et al. demonstrated an elevated LV mass index in KD patients compared to controls and attributed this finding to myocardial edema [24]. We hypothesized that more edematous hearts would function poorly and therefore patients with depressed myocardial mechanics would have higher LV mass index along with increased systemic inflammation. Unexpectedly, in our study, patients with higher strain at baseline showed higher mass indexes compared to those with lower strain. By convalescence, the difference in mass index disappeared and, on average, mass had returned to normal for both groups. Also, in our cohort, we were not able to validate findings by Yu et al. of an association between albumin and either LV mass index or LV strain. Taken together these findings suggest that either myocardial edema is not directly associated with the degree of depressed myocardial strain or that our markers (decreased albumin/increased LV mass index) may not adequately represent the degree of myocardial edema. Further study including imaging modalities capable of direct assessment of myocardial edema is necessary to clarify this question.

Our study was designed to provide insight into the etiology and recoverability of abnormal cardiac mechanics in KD. Beyond mechanistic information, the clinical utility of echo-based determination of myocardial mechanics assessment in KD is evolving. As a tool to aid in the diagnosis of KD, the incidence of low myocardial strain is not high enough to be used as a primary diagnostic criterion for KD. Low strain using the best available cut-off of −18% was, however, present more frequently than coronary artery dilation in our cohort, and might be useful as secondary criteria in difficult diagnostic cases where coronary artery involvement is not present on the initial echo. Additional studies are necessary to understand the incidence of depressed strain in KD compared to other acute systemic inflammatory diseases and its sensitivity and specificity for the diagnosis of KD. For the subset of patients who develop hemodynamic instability, knowledge of abnormal myocardial mechanics could be useful in guiding therapy.

## Limitations

Our study has the standard limitations associated with retrospective studies. As such, we could evaluate associations but could not make definitive conclusions about causality. Additionally, echocardiographic images collected at the time of diagnosis were retrospectively analyzed, and thus were not optimized for post-hoc speckle tracing or for more advanced evaluation of ejection fraction such as Simpson’s Biplane or 3D ejection fraction. Future prospective studies should include imaging protocols focused on more advanced techniques to evaluate myocardial function. Further, the requirement of a serum sample for post-hoc PCT analysis limited the study size and power resulting in an increased likelihood of Type 2 errors especially when further subdividing the patients with lower strain. Also no serum was available to analyze biomarkers of myocardial ischemia or ventricular overload so although we did not find evidence of a relationship between myocardial deformation and proximal coronary changes, we cannot rule out microvascular inflammation/occlusion and resulting ischemia as a cause of poor deformation. Future prospective studies should include measurement of these biomarkers in order to better assess the etiology of depressed strain in KD patients. There was heterogeneity among our patient population including patient age, day of illness at presentation, complete v. incomplete KD, and severity of disease; sub-analyses were not possible due to the limited sample size. Normative data for strain and strain rate are limited. Thus, it is possible that some patients were inappropriately assigned to have “lower” or “higher” deformation based both on limited prior knowledge and the expected bell curve distribution of true biological norms. There was also an incomplete overlap between those patients with abnormal longitudinal and lower circumferential strain. Our study was not sufficiently powered to allow a meaningful subgroup analysis comparing those with entirely preserved mechanics to those with either precisely one or two dimensions of abnormal strain. These subgroups should be analyzed on future prospective studies.

## Conclusions

In this study, the range of strain found in a cohort of acute KD patients was large and likely represented a mixture of depressed, normal, and hyperdynamic function. Improvement in the average strain in the cohort appears to be driven primarily by patients with initially lower strain. Lower strain was associated with increased inflammation, but not proximal coronary artery involvement or elevated LV mass index. Further research is needed to assess the utility of strain as a secondary diagnostic criterion for KD as well as its role in managing hemodynamic instability in critically ill KD patients.

## Figures and Tables

**Table 1:** Demographics and Baseline Information.

Age in months, Mean (SD)	46.3 (32.0)
Sex, n (%)	
Female	20 (48.8)
Male	21(51.2)
Race, n (%)	
White	24 (58.5)
Black	1(2.4)
Hispanic	10 (24.4)
Asian	4 (9.8)
Unknown/Other	2 (4.8)
Days of Illness, Median (IQR)	6 (5,7)
Complete Kawasaki, n (%)	29 (71)
Left Ventricular Mass Index, Mean (SD)	56.3 (15.0)
Ejection Fraction, Mean (SD)	69.1 (6.7)
Abnormal Left Anterior Descending Z-score, n (%)	3 (7.3)
Abnormal Left Main Coronary Artery Z-score, n (%)	2 (5)
Abnormal Right Coronary Artery Z-score, n (%)	4 (9.8)
Peak longitudinal ε, Median (Range)	−18.8% (−28.0, −12.8)
Peak circumferential ε, Median (Range)	−21.2% (−34.6, −11.4)
Peak longitudinal systolic SR, Median (Range)	−1.6%/s (−2.7, −1.0)
Peak circumferential systolic SR, Median (Range)	−2.1%/s (−3.3, 1.0)

**Table 2:** Comparison of markers of inflammation and coronary artery dilation between patients with ε ≤ −18% versus >−18%.

	≤ −18% (n=25)	>−18% (n=16)	P-value
**PCT [ng/mL]**	0.3 (0.1, 25.28)	1.2 (0.12,143.9)	<0.05
**CRP [mg/dL]**	4.9 (0.1, 29.1)	10.9 (1.1,22.6)	NS
**Albumin [g/dL]**	3.55 (2.3, 4.1)	3.3 (2.0, 4.0)	NS
**ESR [mm/h]**	70.4 (29,7)	64.3 (30.9)	NS
**LAD Z-score**	−0.2 (−2.78, 7.78)	0.1 (−1.96, 2.91)	NS
**LMCA Z-score**	−0.1 (−2.36, 4.53)	−0.1 (−1.66, 1.9)	NS
**RCA Z-score**	−0 (−1.94, 2.43)	0.2 (−1.63,2.16)	NS

NS=Non-Significant. Values are presented as median (range) except ESR, which is presented as mean (standard deviation). PCT: Procalcitonin; CRP: C-Reactive Protein; LAD: Left Anterior Descending Coronary Artery; LMCA: Left Main Coronary Artery; RCA: Right Coronary Artery.

**Table 3:** Inflammatory markers in patients with severely depressed longitudinal ε.

	Preserved (n=25)	Mildly Depressed (n=11)	Severely Depressed (n=4)	P-value
PCT [ng/mL]	0.3 (0.1, 25.28)	0.8 (0.12, 9.35)	4.1 (0.13, 143.9)	0.07
CRP [mg/dL]	4.9 (0.1, 29.1)	10.0 (1.10, 22.60)	13.5 (11, 19.1)	0.07

Preserved strain refers to patients with ε ≤ −18%, mildly depressed ε includes patients with −15 %< ε <−18%, and severely depressed ε refers to those with values >−15%. NS: Non-Significant. Values are presented as median (range). PCT: Procalcitonin; CRP: C-Reactive Protein.

**Table 4:** Comparison of markers of inflammation and coronary artery dilation between patients with high and low circumferential ε.

	High (n=29)	Low (n=10)	P-value
**PCT [ng/mL]**	0.4 (0.10, 143.9)	1.8 (0. mg/ml 0, 9.35)	< 0.05
**CRP [mg/dL]**	5.2 (0.10, 29.10)	10 (0.80, 19.10)	NS
**Albmin [g/dL]**	3.4 (2.0, 4.1)	3.55 (2.4, 3.8)	NS
**ESR [mm/h]**	68.9 (31.8)	65 (27.1)	NS
**LAD Z-score**	0 (−2.78, 7.78)	0 (−2.20, 1.36)	NS
**LMA Z-score**	−0.1 (−2.36, 4.53)	−0.1 (−1.47, 1.00)	NS
**RCA Z-score**	−0.1 (−1.66, 4.53)	0 (−1.19, 0.85)	NS

High strain refers to patients with the highest 75% of values; Low strain includes patients with the lowest 25% of values. NS refers to p-values >0.05. Values are presented as median (range) except ESR which is presented as mean (standard deviation). PCT: Procalcitonin; CRP: C-Reactive Protein; LAD: Left Anterior Descending Coronary Artery; LMCA: Left Main Coronary Artery; RCA: Right Coronary Artery.
